# Merkel Cell Carcinoma in the Setting of Chronic Lymphocytic Leukemia and Diffuse Large B-Cell Lymphoma

**DOI:** 10.7759/cureus.17204

**Published:** 2021-08-15

**Authors:** Samaa Alkhouri, Omar Afify, Faris Alkhouri, Hashem Boalbanat, Pragnesh Patel

**Affiliations:** 1 Dermatology, Wayne State University School of Medicine, Detroit, USA; 2 Dermatology, Oakland University William Beaumont School of Medicine, Rochester, USA; 3 Radiology, Wayne State University School of Medicine, Detroit, USA; 4 Internal Medicine, Wayne State University School of Medicine, Detroit, USA

**Keywords:** merkel cell carcinoma, chronic lymphocytic leukemia/small lymphocytic lymphoma, skin cancers, maxillofacial radiology, diffuse large b-cell lymphoma

## Abstract

Merkel cell carcinoma (MCC) is a rare, rapidly growing, and highly malignant cutaneous tumor that typically presents in elderly males as an erythematous or violaceous plaque or nodule in sun-exposed areas. Risk factors include long-term ultraviolet (UV) exposure, Merkel cell polyomavirus (MCV) infection, immunosuppression, and lymphoproliferative disorders such as chronic lymphocytic leukemia/small lymphocytic lymphoma (CLL/SLL). Given the aggressive nature of this tumor, patients may present with nodal and distal metastasis. Locoregional disease can be managed with definitive radiotherapy or surgery with or without adjuvant radiotherapy, depending on the case. Disseminated disease, on the other hand, often requires a multidisciplinary tumor board consultation to individually tailor the treatment. Possible treatments include systemic therapy with chemotherapy or immunotherapy, radiotherapy, and surgery. Here we report a case of a patient with a medical history significant for chronic lymphocytic leukemia and diffuse large B-cell lymphoma who presented with a rapidly growing lesion that contained neighboring MCC and CLL/SLL on biopsy. Management included immunotherapy with pembrolizumab and radiotherapy to limit the tumor's growth and spread. To the best of our knowledge, the coexistence of all three malignancies in a person is rare and has not been reported previously.

## Introduction

Merkel cell carcinoma (MCC) is a rare and aggressive neuroendocrine cutaneous malignancy that has had a rising incidence over the last few decades in the United States (US) [1]. MCC commonly occurs in elderly white males in their seventies or eighties with a history of long-term ultraviolet (UV) exposure. Patients often present with an erythematous or violaceous nodule or plaque in a sun-exposed area, typically in the head or neck [2]. MCC has a high metastatic potential, leading to high rates of recurrence and nodal metastasis. Surveillance and early treatment in certain populations can be life-saving [3].

Patients with autoimmune disease, HIV/AIDS, organ transplants, immunosuppression, or lymphoproliferative disorders are more susceptible to MCC [2]. One of these lymphoproliferative disorders, chronic lymphocytic leukemia/small lymphocytic lymphoma (CLL/SLL) is closely associated with MCC [4]. CLL is a disease that typically affects the elderly and leads to a proliferation of incompetent lymphocytes, compromising a person’s immune response. Major complications of CLL include infections and the development of secondary malignancies including MCC [5]. The coexistence of MCC and CLL is well-documented, and the relationship between MCC and lymphoid malignancies continues to be investigated [4]. 

## Case presentation

A 64-year-old African-American female with a two-year history of CLL and diffuse large B-cell lymphoma (DLBCL), managed only by observation, presented to the emergency department (ED) from her nursing home with a growing, pruritic facial mass on her right cheek. The mass was noticed a month before presentation as a small, painless boil that grew quickly. On exam, a 4 x 3 cm firm, tender bullae with surrounding fluctuance, no erythema, and no warmth was appreciated. No lymphadenopathy was noted. A bedside ultrasound was done and showed a hypoechoic lesion. The patient underwent an incision and drainage, and a small amount of serosanguineous fluid was expressed. A possible dermatologic malignancy was suspected at that time, and the patient was instructed to follow up with a dermatologist after her discharge. However, the coronavirus disease 2019 (COVID-19) pandemic made it difficult for her to make an outpatient appointment, so she did not follow up. 

**Figure 1:**
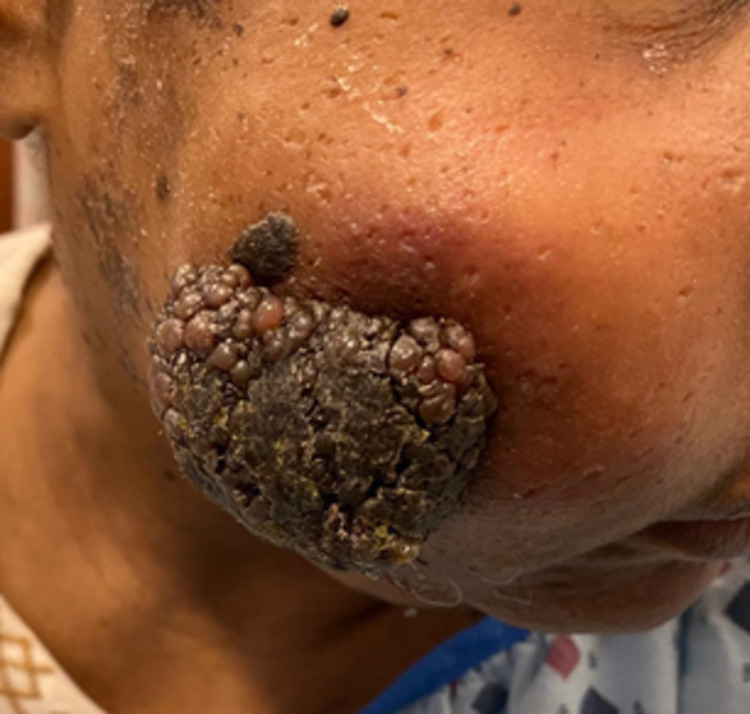
A 5 x 6 cm tender, immobile, hyperpigmented, fungating lesion measuring with a cobblestone appearance on the right cheek

Three months later, the patient returned to the ED with a painless, enlarging mass (Figure 1) in the same location. On exam, a 5 x 6 cm slightly tender, immobile, hyperpigmented, fungating lesion with a cobblestone appearance was appreciated on the right cheek. Non-mobile submandibular, submental, anterior cervical, and medial supraclavicular lymph nodes were noted on the right.

**Figure 2:**
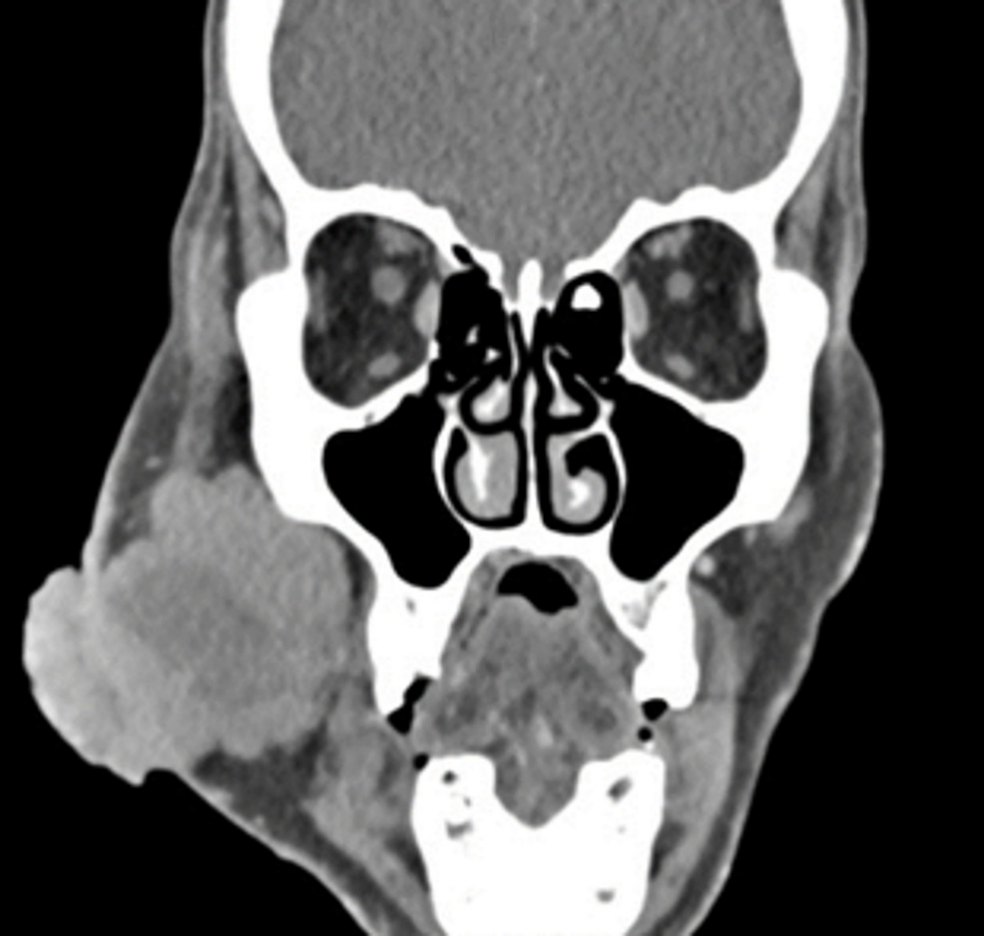
CT scan of the Merkel cell carcinoma, coronal view

**Figure 3:**
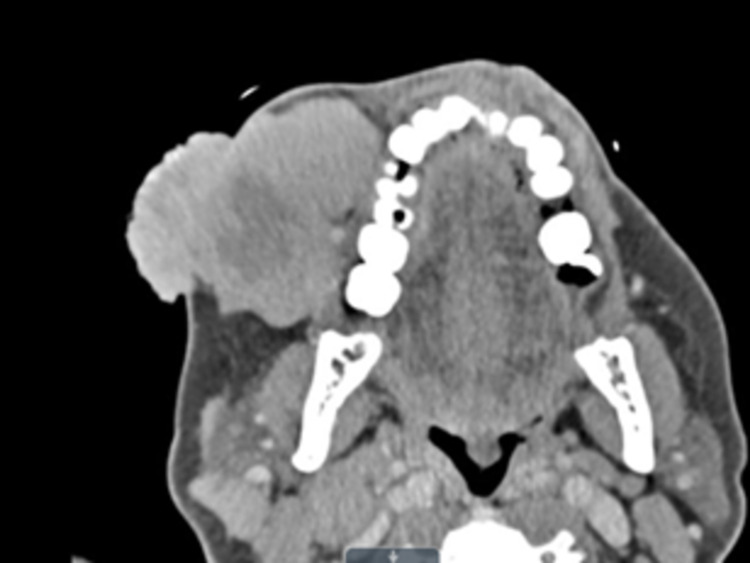
CT scan of the Merkel cell carcinoma, axial view

A computed tomography (CT) scan of the maxillofacial structures with intravenous contrast showed a 5.3 x 6.3 x 5.3 cm soft tissue mass with areas of central necrosis and a superficial polypoid lesion within the right side of the face (Figures 2, 3). The mass abutted the mandible and maxilla but did not appear to cause any osseous invasion nor extend into the buccal cavity or pharyngeal spaces. Unilateral, right-sided, enlarged level 1 and 2 lymph nodes were also appreciated.

Histologically, the biopsy revealed two populations of cells. One population consisted of small- to medium-sized cells with salt-and-pepper chromatin, scant cytoplasm, and hyperchromasia. These cells were positive for synaptophysin, chromogranin, CD56, CAM 5.2, and Tdt. They were negative for P63, Ber-EP4, HMB-45, S100, CD68, and CK7. Cytokeratin-20 (CK20) staining showed focal positivity in a few cells. The Ki-67 index was more than 80%. These findings were consistent with a neuroendocrine carcinoma, favoring MCC.

Moreover, the biopsy sections showed scattered peritumoral lymphoid aggregates composed of small lymphocytes with atypical morphology. The atypical lymphocytes were positive for CD20, PAX5, CD5, CD43, CD45, CD23, and BCL6 (in a partial subset). They were negative for CD3, CD10, MUM1C-MYC, Cyclin D1, and CD99. These findings were consistent with CLL/SLL. Furthermore, no evidence of transformation to DLBCL was identified in the specimen.

Following the biopsy, a multidisciplinary tumor board evaluated our patient for the management of the MCC. Due to the tumor’s location and depth, surgical intervention was not appropriate as it would significantly increase the patient’s morbidity. Since the MCC was nonresectable, immunotherapy with the checkpoint inhibitor pembrolizumab and radiotherapy were initiated.

Two months later, a positron emission tomography (PET)/CT scan showed approximately an 8-cm right buccal mass, which was consistent with the patient's known MCC, metastatic right cervical level 1 through 5 lymph nodes, and likely metastatic left cervical level 2 through 4 lymph nodes.

After six months of treatment, the patient had received four cycles of pembrolizumab and radiation therapy with a planned total dose of 66 Gy to the head and neck. The patient received 33 fractions (Fx) at a rate of 2 Gy/Fx for a total radiotherapy dose of 66 Gy. She tolerated the therapy well with no complications, and the tumor decreased in size to 2 cm in the maximum dimension. 

## Discussion

MCC is a rare and highly malignant primary cutaneous cancer that commonly affects the elderly and those with immunosuppression, exposure to UV, and lymphoproliferative disorders [6]. Typically, MCC presents in Caucasian males in their 70s and 80s as a violaceous or erythematous, tender, indurated, plaque or nodule on the head, neck, or other areas with extensive sun exposure. MCC lesions can also present as telangiectatic papules, cysts, and ulcers. Mucosal involvement has also been documented and is often more aggressive [2]. The pathogenesis of these lesions frequently involves a Merkel cell polyomavirus (MCV) infection. The presence of MCV in human skin is not uncommon, as MCV uses the cell nucleus for replication. In 2008, however, Feng et al. discovered while investigating MCC and MCV that viral integration into the cell genome occurred before clonal expansion, suggesting an important role in tumorigenesis [7].

MCC is uncommon, with only 2500 cases per year in the US; however, the incidence has increased over the last 30 years [1]. Since the 1990s, the incidence has grown by 5-10% per year. Improved detection of MCC with CK20 immunohistochemical staining starting in 1992 likely spurred this increase in incidence [2]. Identification of this frequently misdiagnosed cancer is crucial due to its aggressive nature. MCC grows quickly, and rapid metastasis is common: 26-36% of patients present with nodal metastasis, and 6-16% present with distant metastasis. Unfortunately, metastatic disease is associated with a poor prognosis [6]. The five-year survival is 51% for local lesions, 35% for disease with nodal involvement, and 14% for distant metastatic MCC at presentation [2].

UV exposure and immunosuppression predispose an individual to develop MCC. Eighty-one percent of primary MCC lesions occur in sun-exposed areas, predominately on the head and neck (29-48% of cases) [6]. Moreover, those who received psoralen plus UVA photochemotherapy for psoriasis were 100 times more likely to develop MCC compared to the general population [2]. MCC incidence is also higher in HIV patients, organ transplant recipients, patients with MCV infection, and those with lymphoproliferative disorders such as CLL [2,6]. The coexistence of MCC and CLL has been reported in several publications, and the relationship continues to be investigated [4]. Koljonen et al. documented that the standardized incidence ratio (SIR) for CLL following presentation with MCC was significantly high at 17.9 (95% confidence interval (CI), 2.2-64.6; P<0.001). Furthermore, patients with CLL were more likely to have or develop MCC with a SIR of 15.7 (95% CI, 3.2-46.0; P<0.01) [8].

Diagnostic evaluation of MCC includes biopsy of the lesion and biopsy/aspiration of any palpable nodes. For patients with absent clinical nodes, sentinel lymph node (SNL) biopsy is the most reliable tool for staging [6,9]. Metastatic MCC requires further imaging using CT or PET/CT depending on availability [2,6,9,10]. Initial management of the primary tumor can include several modalities to address the locoregional disease. The National Comprehensive Cancer Network (NCCN) currently recommends wide local excision if clinically feasible with 1- to 2-cm margins to the investing fascia of the muscle or pericranium [6,9]. Adjuvant radiotherapy following excision is also recommended as it improves regional control and outcomes [6,9,11]. The NCCN recommends a radiotherapy dose of 50-66 Gy, depending on the margins [6,9]. Moreover, prophylactic adjuvant radiotherapy to regional nodes can be considered as Jouary et al. have demonstrated that it decreases the probability of regional recurrence; however; no improvement in survival was observed in their study [12].

In a subset of patients, particularly those who are poor surgical candidates or are not amenable to surgery, definitive radiotherapy is recommended [6,9,11,13]. Radiation monotherapy is a viable alternative to surgery and has produced good outcomes in select patients at many Australian institutions, where MCC is more common [11,13]. The current recommendations for definitive radiotherapy from the NCCN are a total dose of 50-66 Gy depending on lymph node biopsy and node dissection status [6,9]. For disseminated disease, a multidisciplinary tumor board consultation is necessary to tailor treatment to the patient's circumstances [6,9]. Possible management modalities include chemotherapy, immunotherapy, radiotherapy, and surgery. Notably, the use of checkpoint inhibitor immunotherapy has emerged recently as a promising option for disseminated disease as it has similar response rates compared with chemotherapy and better durability of response [6,9]. Avelumab, an anti-PD-L1 agent, and pembrolizumab and nivolumab, the anti-PD-1 agents, have shown encouraging preliminary results in treating advanced MCC in current clinical trials [6,9]. In particular, because of its response rate and duration of response, avelumab is the only agent, so far, that has received accelerated FDA approval for all patients (12 years and older) with metastatic MCC [6].

Our patient’s presentation is rare for several reasons. First, our patient is an African American female, and while MCC has been reported in patients of other ethnicities and backgrounds, it commonly occurs in white males. Second, based on our literature review, this is the first case of a patient having CLL, DLBCL, and MCC at once. Moreover, the biopsy of the lesion was remarkable for the coexistence of MCC and CLL/SLL. While this finding is not unique, it warrants an investigation into the causative agents linking the two diseases [4,14].

Finally, our case highlights the risk of developing secondary cutaneous malignancies even if the lymphoproliferative disorder does not require treatment. This case emphasizes the importance of skin cancer screening for patients with CLL and/or DLBCL. The COVID-19 pandemic prevented our patient from visiting a dermatologist, which could have changed the patient’s morbidity significantly if the MCC was caught early [2]. Dermatologists conducting annual skin exams should have MCC on their differential diagnosis for patients with suspicious lesions in sun-exposed areas and a history of lymphoproliferative disorders.

## Conclusions

Our case reports a 64-year-old female with a medical history significant for CLL and DLBCL who presented with an enlarging lesion that contained MCC and CLL/SLL. MCC is a rare and aggressive cutaneous malignancy that commonly occurs in sun-exposed areas and in individuals with immunosuppression and hematological malignancies such as CLL. Our patient’s cancer grew rapidly and was unresectable due to its depth and location. Immunotherapy and palliative radiation were used to limit the tumor's growth and spread. Patients with CLL, DLBCL, or other lymphoproliferative disorders should be carefully followed and screened for secondary cutaneous malignancies in their early stages to improve overall morbidity and mortality. 

## References

[1] Hodgson NC (2005). Merkel cell carcinoma: changing incidence trends. J Surg Oncol.

[2] Coggshall K, Tello TL, North JP, Yu SS (2018). Merkel cell carcinoma: an update and review: pathogenesis, diagnosis, and staging. J Am Acad Dermatol.

[3] Harms KL, Healy MA, Nghiem P, Sober AJ, Johnson TM, Bichakjian CK, Wong SL (2016). Analysis of prognostic factors from 9387 Merkel cell carcinoma cases forms the basis for the new 8th Edition AJCC staging system. Ann Surg Oncol.

[4] Tadmor T, Aviv A, Polliack A (2011). Merkel cell carcinoma, chronic lymphocytic leukemia and other lymphoproliferative disorders: an old bond with possible new viral ties. Ann Oncol.

[5] Tsimberidou AM, Wen S, McLaughlin P (2009). Other malignancies in chronic lymphocytic leukemia/small lymphocytic lymphoma. J Clin Oncol.

[6] Bichakjian CK, Olencki T, Aasi SZ (2018). Merkel cell carcinoma, version 1.2018, NCCN clinical practice guidelines in oncology. J Natl Compr Canc Netw.

[7] Feng H, Shuda M, Chang Y, Moore PS (2008). Clonal integration of a polyomavirus in human Merkel cell carcinoma. Science.

[8] Koljonen V, Kukko H, Pukkala E (2009). Chronic lymphocytic leukaemia patients have a high risk of Merkel-cell polyomavirus DNA-positive Merkel-cell carcinoma. Br J Cancer.

[9] Schmults C, Blitzblau R, Aasi S (2019). NCCN clinical practice guidelines in oncology for Merkel cell carcinoma, version 2.2019. J Natl Compr Canc Netw.

[10] Pape E, Rezvoy N, Penel N (2011). Radiotherapy alone for Merkel cell carcinoma: a comparative and retrospective study of 25 patients. J Am Acad Dermatol.

[11] Kok DL, Wang A, Xu W (2020). The changing paradigm of managing Merkel cell carcinoma in Australia: an expert commentary. Asia Pac J Clin Oncol.

[12] Jouary T, Leyral C, Dreno B (2012). Adjuvant prophylactic regional radiotherapy versus observation in stage I Merkel cell carcinoma: a multicentric prospective randomized study. Ann Oncol.

[13] Gunaratne DA, Howle JR, Veness MJ (2017). Definitive radiotherapy for Merkel cell carcinoma confers clinically meaningful in-field locoregional control: a review and analysis of the literature. J Am Acad Dermatol.

[14] Saade R, Najjar S, Arslan ME, Rady P, Tyring SK, Nazeer T (2021). Concurrent adjacent Merkel cell carcinoma and chronic lymphocytic leukemia without simultaneous Merkel cell polyomavirus detection: a case series. Dermatopathology (Basel).

